# Intra-decadal increase in globally-spread *Magallana gigas* in southern California estuaries

**DOI:** 10.1371/journal.pone.0302935

**Published:** 2024-05-08

**Authors:** Marah L. Wolfe, Chelsea M. Bowers-Doerning, Anabell Espinosa, Ty Frantz, William J. Hoese, Joann G. Lam, Kailee R. Lamp, Rachael A. Lyons, Justin K. Nguyen, Bryce D. Keyes, Jada Smith, Holly L. Suther, Meaghan Swintek, Juliann C. Vannordstrand, Danielle C. Zacherl

**Affiliations:** 1 Department of Biological Science, California State University Fullerton, Fullerton, California, United States America; 2 College of the Environment, Western Washington University, Bellingham, WA, United States America; Bigelow Laboratory for Ocean Sciences, UNITED STATES

## Abstract

Introduction and establishment of non-indigenous species (NIS) has been accelerated on a global scale by climate change. NIS *Magallana gigas’* (formerly *Crassostrea gigas’*) global spread over the past several decades has been linked to warming waters, specifically during summer months, raising the specter of more spread due to predicted warming. We tracked changes in density and size distribution of *M*. *gigas* in two southern California, USA bays over the decade spanning 2010–2020 using randomly placed quadrats across multiple intertidal habitats (e.g., cobble, seawalls, riprap) and documented density increases by 2.2 to 32.8 times at 7 of the 8 sites surveyed across the two bays. These increases in density were coincident with 2–4° C increases in median monthly seawater temperature during summer months, consistent with global spread of *M*. *gigas* elsewhere. Size frequency distribution data, with all size classes represented across sites, suggest now-regular recruitment of *M*. *gigas*. Our data provide a baseline against which to compare future changes in density and abundance of a globally-spread NIS of significant concern.

## Introduction

Climate change has accelerated the introduction and establishment of non-indigenous species (NIS) on a global scale [1]. Range expansions of warm-adapted species have been recorded on every continent, creating novel interactions among species within the recipient communities [2, 3]. Additionally, anthropogenic responses to climate change may unintentionally favor NIS. For instance, the construction of coastal infrastructure to counter rising sea levels and erosion can create habitats that may favor the recruitment and spread of NIS [4, 5]. Simultaneously, sea surface temperatures (SSTs), are projected to rise between 0.25–0.50°C per decade [6] and are positively correlated with stress and mortality in some native marine species [7, 8] while rapid shifts in seasonal temperatures can favor NIS timing of recruitment [9]. As native species are unable to compete or are no longer physically present to occupy the niche they once held [10], their communities become more susceptible to colonization by NIS. Success of initial NIS can facilitate the success of future invaders, resulting in large scale community shifts dominated by NIS [11, 12], posing a significant threat to the persistence of native communities [13].

The Pacific oyster, *Magallana gigas* [formerly *Crassostrea gigas;* 14], is a NIS whose global expansion may have been facilitated by climate change. *Magallana gigas* is native to Japan but was introduced to several countries as an aquaculture fishery [15], including into the USA along the Pacific coast of North America following the collapse of the native Olympia oyster (*Ostrea lurida*) fishery [16]. *Magallana gigas* was introduced as an ideal aquaculture fishery into some cold-temperate waters under the premise that it could successfully grow in cold waters but could not complete its life cycle [17] and thus posed little risk for feral escape. However, warming seawater during summer has facilitated the spawning and increased recruitment of *M*. *gigas* [18] into regions outside its historical range along European coasts [19–21]. Based on projected rises in seawater temperature, *M*. *gigas* is expected to expand its range poleward along the Northwest European Shelf, with the majority of the coastline experiencing suitable thermal conditions for successful recruitment by 2100 [22]. These range expansions have not been limited to European coasts; *M*. *gigas* have been introduced to every continent aside from Antarctica [16]. In North America, increases in *M*. *gigas* recruitment since 1945 are strongly correlated with warming waters [23] and have been observed in estuaries across British Columbia, the U.S. West Coast, and Baja California, Mexico [24].

While evidence for *M*. *gigas* range expansion in response to climate change is strong, the ecological impact on recipient communities is less clear, ranging from positive to negative. Their impact appears to be dependent on substrate type, the amount of physiological stress experienced by recipient community members, and whether native engineering species are already present within the affected ecosystem [16]. *Magallana gigas* modify existing habitats through gregarious settlement; they cement their shells together and can create a reef [25]. By providing vertical relief [26] and microhabitats where larvae can settle [27], these reefs can facilitate increases in species diversity [25]. In the Oosterschelde estuary in the Netherlands, *M*. *gigas* reefs aid in preventing erosion and, therefore, help to protect native intertidal bivalves and fauna, as well as providing foraging grounds for seabirds [25]. In Patagonia, shorebirds have been found to spend more time foraging in *M*. *gigas* reefs, likely due to higher prey availability [28]. On the other hand, native clams recruiting to *M*. *gigas* reefs experience higher predation than those on native habitats because the *M*. *gigas* reefs supported higher densities of predatory crabs [29]. In the Bay of Brest, France, areas of bare mud were dominated by suspension feeders; however, when *M*. *gigas* transformed the mud into an oyster reef, carnivores became the dominant trophic group, and suspension feeder abundance declined dramatically [20]. *Magallana gigas* have displaced local mussel populations in several European countries [30], often decimating other native organisms in the process. Introduced *M*. *gigas* in Australia rapidly overgrew native oysters at mid and low intertidal heights [31], although the native species escaped competition at higher tidal elevations. In Washington, USA, *M*. *gigas* may cause native *O*. *lurida* to recruit to a higher tidal elevation than is optimal for the species, leading to thermal or desiccation stress for the native oyster [32]. *Magallana gigas* aquaculture in Washington state also introduced a predatory gastropod *Ocinebrellus inornatus* [formerly *Tritonalia japonica*; 33] as well as a parasite, *Mikrocytos mackini*, that may infect *O*. *lurida* [34]. Because *M*. *gigas* has had significant impacts as a NIS, ranging from positive and negative, its introduction and population demographics in new areas should be carefully monitored.

*Magallana gigas* was first introduced to northern California, USA through aquaculture in the 1920s and in southern California a decade later in Newport Bay [35]. Despite multiple intentional plantings of *M*. *gigas* into southern California locations from the 1930s through to the 1980s [35–37], there were no consistent reports of established populations until 2000 when wild *M*. *gigas* observations were recorded first in Los Angeles Harbor and San Diego Bay [38]. This was followed by reports of *M*. *gigas* in Alamitos Bay [39], San Diego Bay and Mission Bay [39, 40], and Tijuana Estuary and Oceanside Harbor [40] with multiple size classes observed across embayments throughout San Diego County indicating recent reproduction [40]. The first quantitative data on *M*. *gigas* densities were reported from San Diego Bay and Newport Bay, California between 2010 and 2013 [41] and established a baseline against which any changes in population density could be compared.

Here, we examine the status of *M*. *gigas* densities in two southern California bays to address the following questions: Have *M*. *gigas* densities increased over the decade since the quantitative baseline was established? Is there evidence of regular recruitment, or is the establishment of this species the result of rare recruitment events? Can putative changes in *M*. *gigas* density be linked to changes in SSTs?

## Methods

### Overview

**Fig 1:**
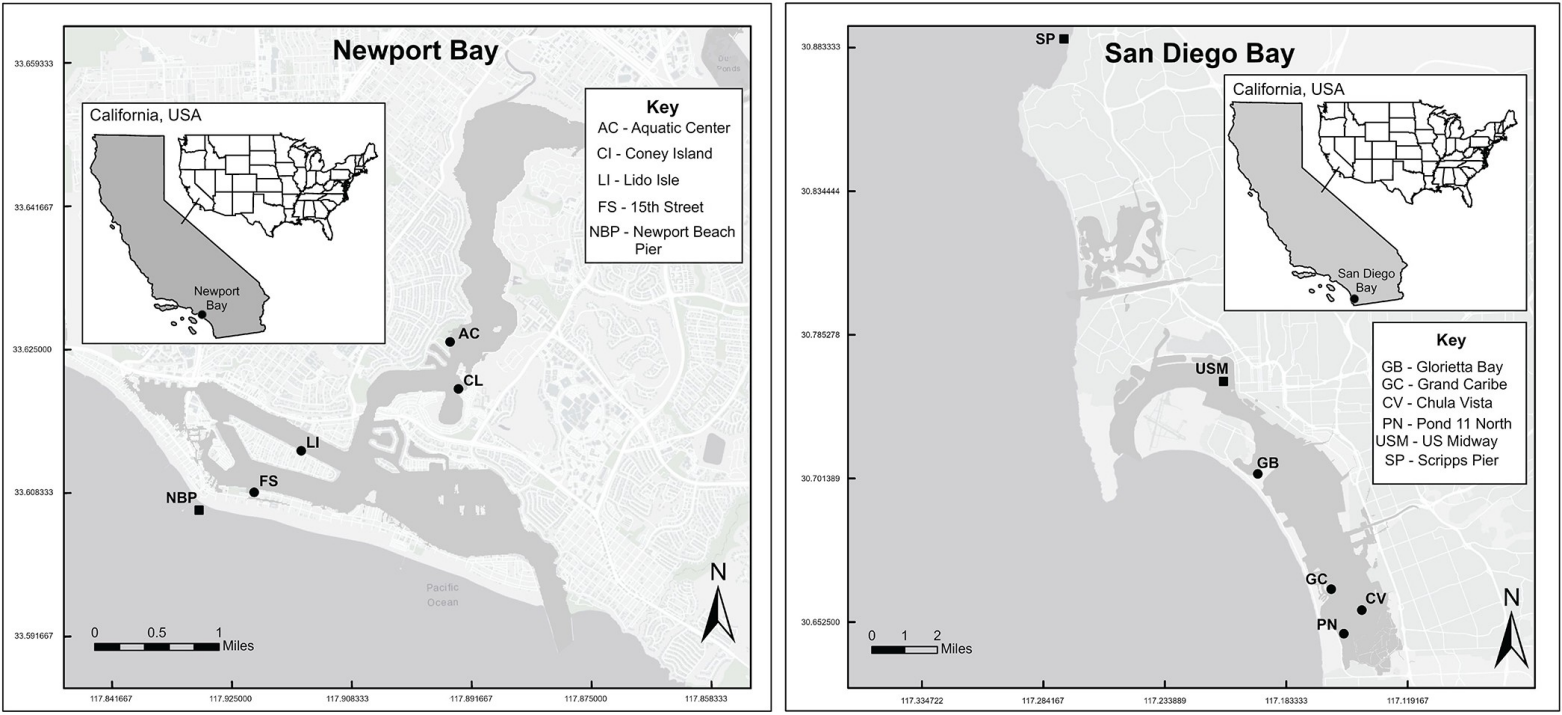
Site locations surveyed from 2010–2020 in Newport Bay (left panel) and from 2013–2020 in San Diego Bay (right panel), California, USA. Squares indicate locations where temperature data were collected and circles reference sites surveyed with site codes as in Table 1. Basemap and service layer content is the intellectual property of Esri and is used herein with permission. Copyright © 2023 Esri and its licensors. All rights reserved.

From 2010 to 2020, we surveyed *M*. *gigas* densities and size frequency distributions at 4 sites each in Newport Bay and San Diego Bay, California, USA (Fig 1, Table 1). Sites were surveyed two to six times throughout the decade (median = 3). The habitats among the sites varied and included pier piles, seawalls, riprap, cobble, mudflat, and chain link fence (Table 1). A California Department of Fish and Wildlife scientific collecting permit (SCP-3027) required to complete this research was issued to author D. Zacherl and all appropriate approvals were obtained for the research.

**Table 1:** Details for Southern California, USA surveys conducted from November 2010 to June 2020, including bay, site names and codes, habitat types, years surveyed, GPS coordinates, tidal range surveyed (m MLLW), tidal range used in statistical comparisons (m MLLW), and *M*. *gigas*/m^2^ with standard error (SE) and sample size (n) included. NOAA Tide Prediction Station for Newport Bay sites was 9410580 and for San Diego sites was 9410152.

Bay	Site	Code	Habitat Type	Year	Date(s) Surveyed	GPS Coord.	Tidal Range Surveyed (m MLLW)		Tidal Range Compared (m MLLW)		*M*. *gigas*/m^2^ (SE, n)
							Min	Max	Min	Max	
Newport	Aquatic Center	AC	Riprap	2012	9-Jan-2012, 8-Feb-2012	33.6234, -117.8934	-0.3	0.65	-0.3	0.65	4.50 (1.6, 30)
			Riprap	2015	25-Nov-2015	33.6234, -117.8935	-0.3	0.55	-0.3	0.55	5.90 (2.2, 13)
			Riprap	2016	24-May-2016	33.6234, -117.8936	-0.49	0.7	-0.49	0.7	5.90 (2.4, 15)
			Riprap	2020	6-Jun-2020	33.6234, -117.8937	-0.11	0.86	-0.11	0.86	12.30 (2.5, 15)
	Coney Island	CI	Natural Cobble	2010	5-Nov-2010	33.6195, -117.8921	-0.12	0.48	-0.12	0.48	0.53 (0.25, 30)
			Natural Cobble	2016	14-Dec-2016	33.6195, -117.8922	-0.17	0.51	-0.17	0.51	2.67 (0.67, 30)
			Natural Cobble	2017	29-May-2017	33.6195, -117.8923	-0.15	0.75	-0.15	0.49	2.27 (0.48, 60)
	15th Street	FS	Pier Piling	2010,2011	6-Nov-2010, 17-Jan-2011	33.6082, -117.9202	-0.01	1.24	-0.01	1.24	1.75 (0.63, 16)
			Seawall	2012	5-Mar-2012	33.6082, -117.9203	-0.02	0.94	-0.02	0.94	1.85 (0.86, 13)
			Seawall	2016	29-Dec-2016	33.6082, -117.9204	-0.02	0.94	-0.02	0.94	8.80 (4.53, 10)
			Seawall	2017	30-May-2017	33.6082, -117.9205	-0.1	0.85	-0.1	0.85	7.59 (1.13, 59)
			Pier Piling	2019	28-Sep-2019	33.6082, -117.9206	0.01	1.07	0.07	1.07	68.25 (7.77, 59)
			Pier Piling	2020	22-Feb-2020	33.6082, -117.9207	-0.23	0.96	-0.23	0.96	57.32 (8.37, 67)
	Lido Island	LI	Seawall/Pier Piling	2011	19-Jan-2011, 20-Jan-2011, 19-Feb-2011	33.6132, -117.9156 33.6115, -117.9119	-0.39	0.78	-0.39	0.78	11.45 (1.26, 44)
			Seawall	2013	29-Apr-2013, 30-Apr-2013	33.6132, -117.9156 33.6115, -117.9120	-0.39	0.78	-0.39	0.78	8.44 (1.50, 27)
			Seawall	2016	7-Jun-2016	33.6132, -117.9156 33.6115, -117.9121	-0.36	0.8	-0.36	0.8	25.28 (3.21, 25)
San Diego	Chula Vista WR	CV	Cobble	2013	28-May-2013	32.6143, -117.1138	0.1	0.38	0.1	0.38	7.00 (3.08, 24)
			Cobble	2017	27-May-2017, 25-Jul-2017	32.6143, -117.1139	-0.3	1.06	0.11	0.59	4.76 (2.48, 37)
			Cobble	2020	8-Jun-2020	32.6143, -117.1140	-0.29	1.81	0.04	0.56	28.33 (4.36, 12)
	Glorietta Bay	GB	Fence	2014	26-May-2014	32.6751, -117.1674	0.2	1.11	0.2	1.11	58.09 (9.69, 23)
			Fence	2017	26-Jul-2017	32.6751, -117.1675	0	1.25	0.2	1.1	150.40 (25.21, 25)
			Fence	2020	10-Jun-2020	32.6751, -117.1676	0.08	1.2	0.22	1.11	222.67 (34.03, 30)
	Grand Caribe	GC	Sand/Riprap	2013	25-Jul-2013	32.6263, -117.1297	0.39	0.93	0.39	0.93	44.13 (8.74, 30)
			Sand/Riprap	2017	13-Jan-2017, 28-May-2017	32.6263, -117.1298	-0.3	0.81	0.3	0.81	34.00 (5.26, 30)
			Sand/Riprap	2020	9-Jun-2020	32.6263, -117.1299	-0.04	1.19	0.37	1.09	35.20 (12.44, 20)
	Pond 11 North	PN	Mud with Concrete Rubble	2013	13-Dec-2013	32.6027, -117.1178	0.15	0.44	0.15	0.44	0.00 (0, 30)
			Mud with Concrete Rubble	2020	23-Jun-2020	32.6027, -117.1179	-0.26	1.01	0.16	0.59	8.35 (4.55, 23)

### Density

All surveys occurred within the intertidal zone during low tide at tidal elevations ranging from -0.49 m to +1.81 m MLLW, although the tidal range surveyed varied across years and sites, with broader ranges of elevations surveyed in more recent years, particularly in San Diego Bay. Standardization of tidal ranges for statistical comparisons among years is addressed below (see Statistical Analyses, Table 1); data recorded at tidal elevations not included in this study are available upon request to the corresponding author.

From 2010–2016, we surveyed replicate randomly placed 0.5 m x 0.5 m (0.25 m^2^) quadrats along a 50-m transect placed in the middle of the visible oyster zone parallel to the water line. We surveyed randomly selected elevations for each quadrat placed within a 1–4 m band above and below the transect line; the band width depended upon the slope of substrate surveyed. Tidal elevation was estimated for the entire transect at each location with RTK-GPS or a LaserMark LM-30, with maximum and minimum elevations recorded (see Table 1 for tidal range surveyed). For pier piles, we did not lay a transect, instead we randomized the pile and side of pile surveyed, and elevation surveyed.

Once we established that tidal elevation played a significant role in oyster distributions [41], we recorded tidal elevation of every randomized quadrat in all subsequent surveys (2017–2020). We also modified sampling on habitats with steep slopes (seawall, pier pile and fence habitats) to more precisely account for tidal elevation by using quadrats sized 0.5 m length X 0.15 m height (0.075 m^2^). These smaller-area quadrats are as long as those used prior to 2017, but not as high, thus sampling a more constrained tidal range on a vertical surface. In 2020, we sampled across a much broader tidal elevation range relative to previous years (e.g., see Table 1 for tidal ranges surveyed) but constrained among-year comparisons of density data to commonly surveyed tidal elevations.

Within each quadrat, we identified and counted all live *M*. *gigas* that were at least 50% inside of the quadrat. We used internal and external shell characteristics to identify *M*. *gigas*. External characteristics that differentiated NIS *M*. *gigas* from native *O*. *lurida* (the only other oyster species present in our surveyed estuaries) included larger maximum height, thicker body, and the presence of shell foliations. Internal characteristics included a chalky white interior shell color and absence of chomata; presence of chomata is a generic-level trait for *Ostrea* [42, 43]; also see Fig 1 of [24]. We recorded the heights and lengths of as many *M*. *gigas* encountered as was practical given low-tide time constraints (n = 31–308 oyster lengths recorded per survey, median = 112); height of each oyster was measured from the umbo to the furthest lip of the oyster shell. When oyster densities were low (i.e., prior to 2015), every oyster encountered was measured. When oyster densities were high (2015 and later) it became impossible to measure all oyster heights within a single tide; surveyors were instructed to systematically work across the quadrat and only measure the heights of the first 5 oysters encountered.

### Size frequency distribution

Oyster shell heights were used to visualize changes in size frequency distributions before and after 2015 as an indicator for recruitment through time. Shell height measurements were recorded at three sites in Newport Bay (FS, LI, and AC), and one site in San Diego Bay (CV). Heights were binned into 10 mm increments (i.e., 10–19 mm, 20–29 mm, etc.) and distributions were compared within each site before and after 2015. Shell heights < 10 mm were excluded. Oyster sizes from recruitment plates in San Diego Bay, deployed yearly from May through September [44] indicate that oysters less than ~40 mm can generally be considered Year 0 (young of the year) and those > 40 mm considered Year 1 or older, though growth is known to vary broadly across sites [45] based upon food availability, temperature and salinity [46].

### Sea surface temperature

We downloaded publicly available sea surface temperature (SST) data from the Shore Stations Program [47] at Newport Beach Pier (open coast near Newport Bay, 33.606414°N, 117.929997°W), Scripps Pier (open coast near San Diego Bay, 32.866692°N, 117.257496°W), and from the National Data Buoy Center [48] for NOAA station IIWC1 at the US Midway (inside of San Diego Bay, 32.713889°N 117.176667°W). All SST temperature data spanned from January 1, 2010, to December 31, 2020. Some SST data were missing from each year at Newport Beach and Scripps Piers, especially during June (18 days) and July (11 days), 2014 at Newport Beach Pier. Temperature data at Newport Beach Pier were recorded once per day sporadically between 06:00–18:00 (Pacific time), at Scripps Pier once per day between 07:00–18:00 (Pacific time), and hourly at NOAA station IIWC1. Since *M*. *gigas* is known to settle and recruit in southern California during summer from June to September [44], we focused our temperature data exploration during those months. We reviewed the daily SST data from 2010 through 2020 to look for a noticeable difference in summer water temperatures that may have correlated with increasing local *M*. *gigas* densities.

### Statistical analyses

To account for discrepancies in tidal elevation sampled among survey years, with some earlier survey years constrained to a smaller subset of elevations surveyed, we standardized the ranges of elevations so that consistent elevations were compared within each site across years, but not necessarily across sites (e.g., elevations surveyed at CV were lower than those at GC, see Table 1 for the full range of sites surveyed versus the standardized range used in statistical analyses). For each site, we examined whether oyster density changed across time using 1-way ANOVA (time treated as categorical factor) in JMP v16.0.0. We did not combine sites into the same statistical analysis because each site had a unique history of years surveyed and standardized elevations that could be used. Most data were log-transformed [Log (X+ 1)] to address heteroscedasticity prior to analysis (Table 2). The log-transformations greatly improved issues with heteroscedasticity and non-normality as was evidenced by visual inspection of plots of residuals by predicted, studentized residuals, and residual by normal quantiles. Statistical analyses were not performed on the SST and size frequency distribution data; qualitative trends were elucidated via visual inspection of the data.

**Table 2:** One-way ANOVA test results and data transformations comparing *M*. *gigas* densities across years for sites within Newport and San Diego Bays, CA, USA. DF = degrees freedom, MS = mean squares, Significant findings bold.

Bay	Site	Transformation	Source	DF	MS	F Ratio	Prob > F
Newport Bay	AC	Log (X+1)	Year	3	7.14099	5.0367	**0.0033**
			Error	69	1.4178		
			C. Total	72			
	CI	Log (X+1)	Year	2	3.4840	4.673	**0.0112**
			Error	117	0.7456		
			C. Total	119			
	FS	Log (X+1)	Year	5	49.1874	17.2692	**< .0001**
			Error	248	2.8483		
			C. Total	253			
	LI	Log (X+1)	Year	2	11.2178	14.4689	**< .0001**
			Error	93	0.7753		
			C. Total	95			
San Diego	GB	None	Year	2	176324	9.2075	**0.0003**
			Error	75	19150		** **
			C. Total	77			** **
	PN	Log (X+1)	Year	1	5.9306	6.6696	**0.0127**
			Error	51	0.8892		** **
			C. Total	52			** **
	CV	Log (X+1)	Year	2	11.6770	6.4669	**0.0026**
			Error	70	1.8056		** **
			C. Total	72			
	GC	Log (X+1)	Year	2	6.9650	2.3795	0.0994
			Error	77	2.9271		
			C. Total	79			

## Results

### Density

**Fig 2:**
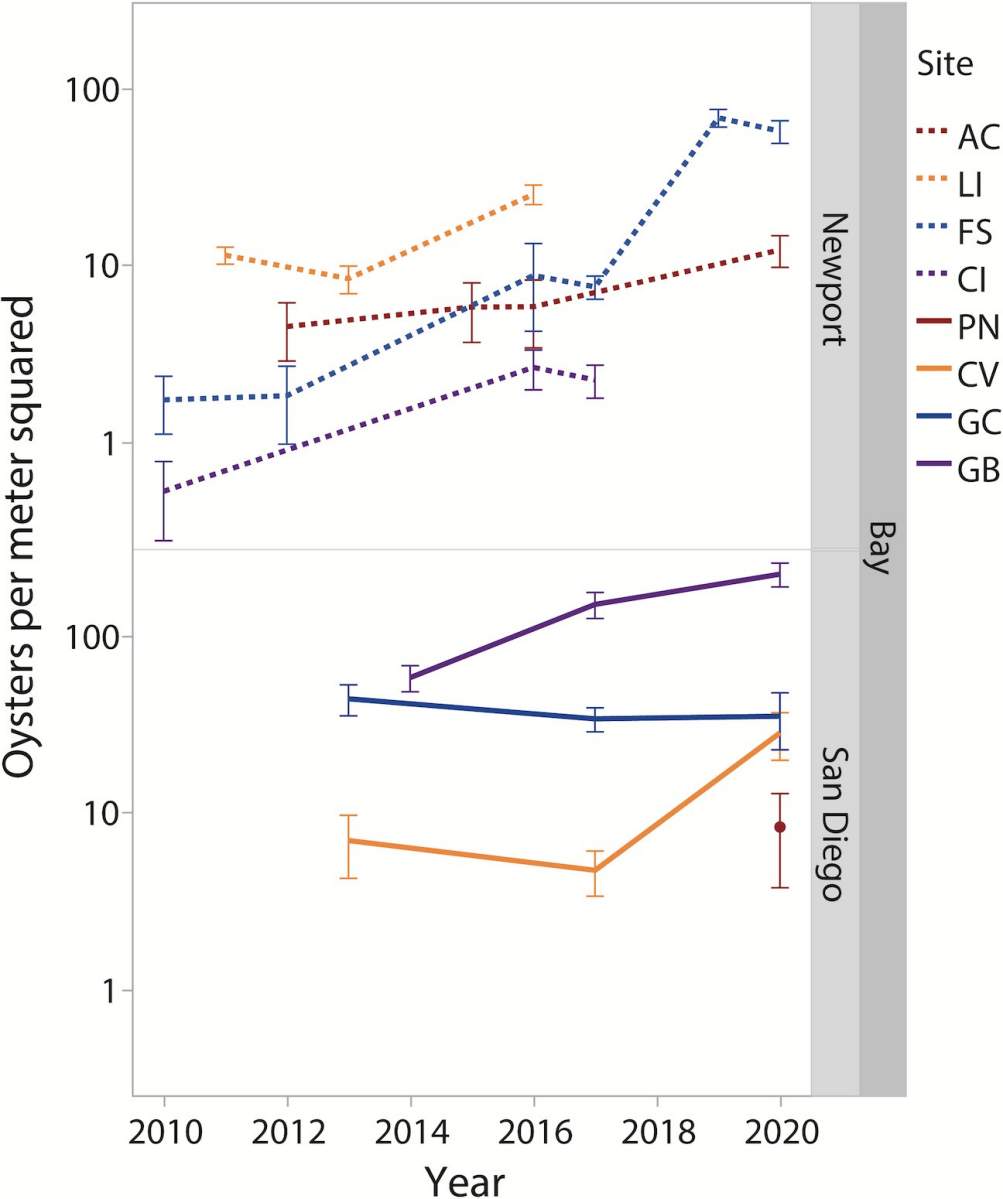
Average *M*. *gigas* densities per m^2^ from 2010–2020 in Newport (upper panel, dashed lines) and San Diego (lower panel, solid lines) Bays, CA, USA. Y-axis is log scale. Site colors reference the relative distance to the mouth of the bay (cooler colors = closer, warmer colors = farther). PN was surveyed in 2013 and *M*. *gigas* was not detected, therefore its 2013 density cannot be graphed on a log scale. Error bars are ± 1 standard error.

From 2010 to 2020, *M*. *gigas* density increased by 2.2 to 32.8 times at 7 of the 8 sites surveyed across two southern California bays (Fig 2).

In Newport Bay, *M*. *gigas* densities significantly increased over time at all 4 sites. At AC, density increased 2.7 times from 4.53 ± 1.64 (SE) to 12.27 ± 2.48 oysters/m^2^ (Tables 1–3, Fig 2, ANOVA, p = 0.0033). At CI, density increased 23.2 times from 0.53 ± 1.64 to 12.27 ± 2.48 oysters/m^2^ (Tables 1–3, Fig 2, p = 0.0112). At LI, density increased 2.2 times from 11.45 ± 1.26 to 25.28 + 3.21 oysters/m^2^ (Tables 1–3, Fig 2, p < 0.0001). At FS, density increased 32.8 times from 1.75 ± 0.63 to 57.32 ± 8.38 oysters/m^2^ (Tables 1–3, Fig 2, p < 0.0001).

**Table 3:** Post-Hoc Tukey-Kramer HSD test results. Letters that are not shared indicate significant differences between years within study sites (Fig 2).

Bay	Site	Year	Letters
Newport Bay	AC	2012	A
		2015	AB
		2016	AB
		2020	B
	CI	2010	A
		2016	B
		2017	B
	FS	2010–2011	A
		2012	A
		2016	A
		2017	AB
		2019	BC
		2020	C
	LI	2010	A
		2013	A
		2016	B
San Diego	GB	2014	A
		2017	B
		2020	B
	P11N	2013	A
		2020	B
	CVWR	2013	A
		2017	A
		2020	B

In San Diego Bay, *M*. *gigas* densities increased over time at 3 of the 4 sites. At PN, density increased from 0.00 ± 0.00 to 8.35 ± 4.55 oysters/m^2^ (Tables 1–3, Fig 2, p = 0.0127). At CV, density increased 4.1 times from 7.00 ± 3.08 to 28.33 ± 4.36 oysters/m^2^ (Tables 1–3, Fig 2, p = 0.0026). At GC, density was stable over time and averaged from 35.20 ± 12.44 to 44.13 ± 8.74 (1 SE) oysters/m^2^ (Tables 1 and 2, Fig 2, p = 0.0994). At GB, density increased 3.8 times from 58.09 ± 9.69 to 222.67 ± 34.03 oysters/m^2^ (Tables 1–3, Fig 2, p = 0.0003).

### Size frequency distributions

**Fig 3:**
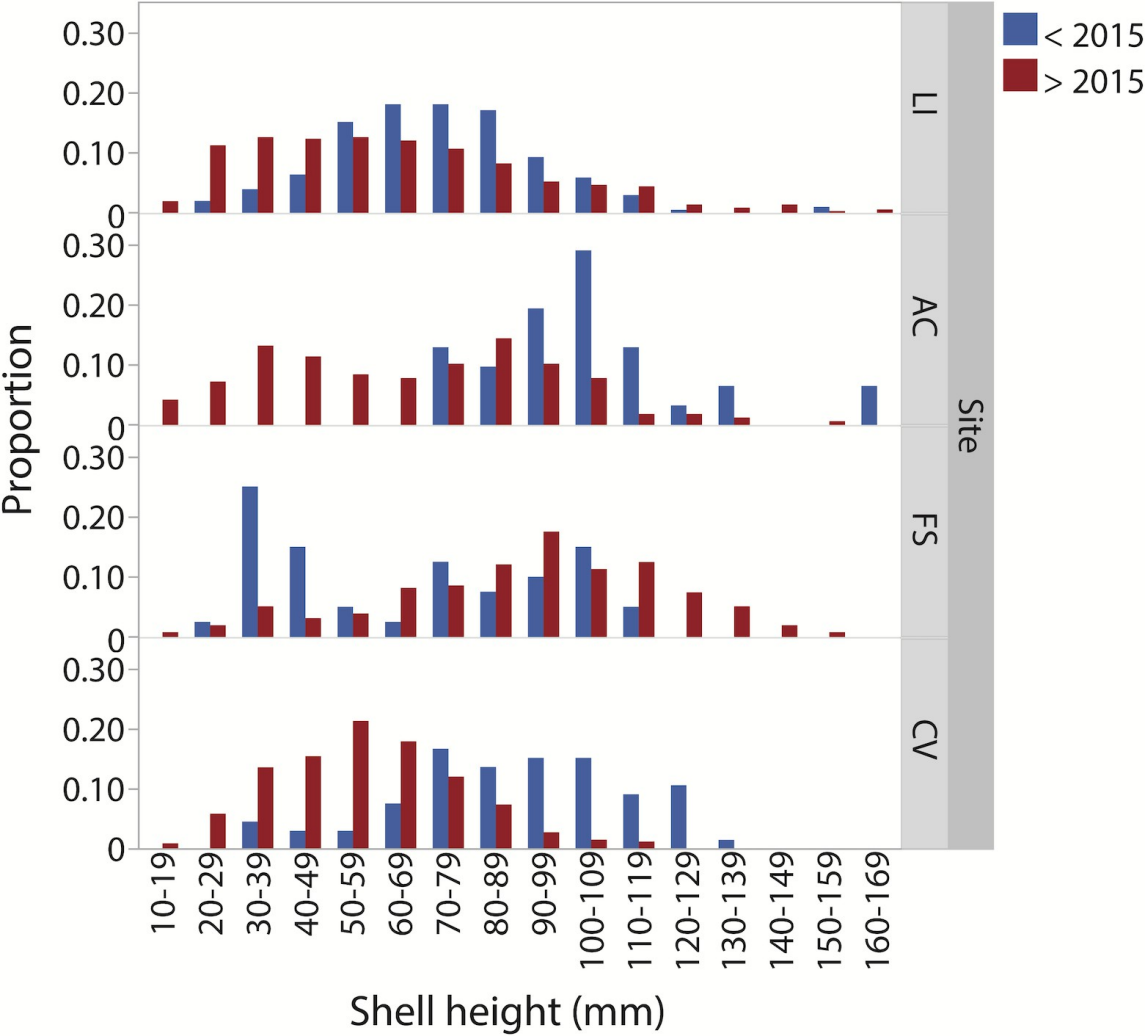
Relative size frequency distributions (proportions) of *M*. *gigas* individuals per 10-mm size bin for the time periods 2010–2015 versus 2016–2020 in Newport (sites LI, AC, FS) and San Diego (CV) Bays, CA, USA.

From 2010 through 2015, *M*. *gigas* in the 10-mm size class, representing very recent Year 0 recruitment, were not detected at the three sites surveyed in Newport Bay nor at CV in San Diego Bay (Fig 3). All sites showed increased proportions of this size class following 2015, ranging between 0.8% at FS and 4.2% at AC. At three of four sites (LI, AC and CV), there were also higher proportions of oysters (6%– 9.3%) in size classes from 20 mm to 40 mm (also likely Year 0) relative to before 2015. Unlike at the other three sites, proportions of oysters in most of the smaller size classes (20–50 mm) at FS in Newport Bay decreased between 0.6% and 19.9% after 2015. All sites showed evidence of Year 0 size classes (< 40 mm, see methods) both before and after 2015 (Fig 3).

### Sea surface temperature

**Fig 4:**
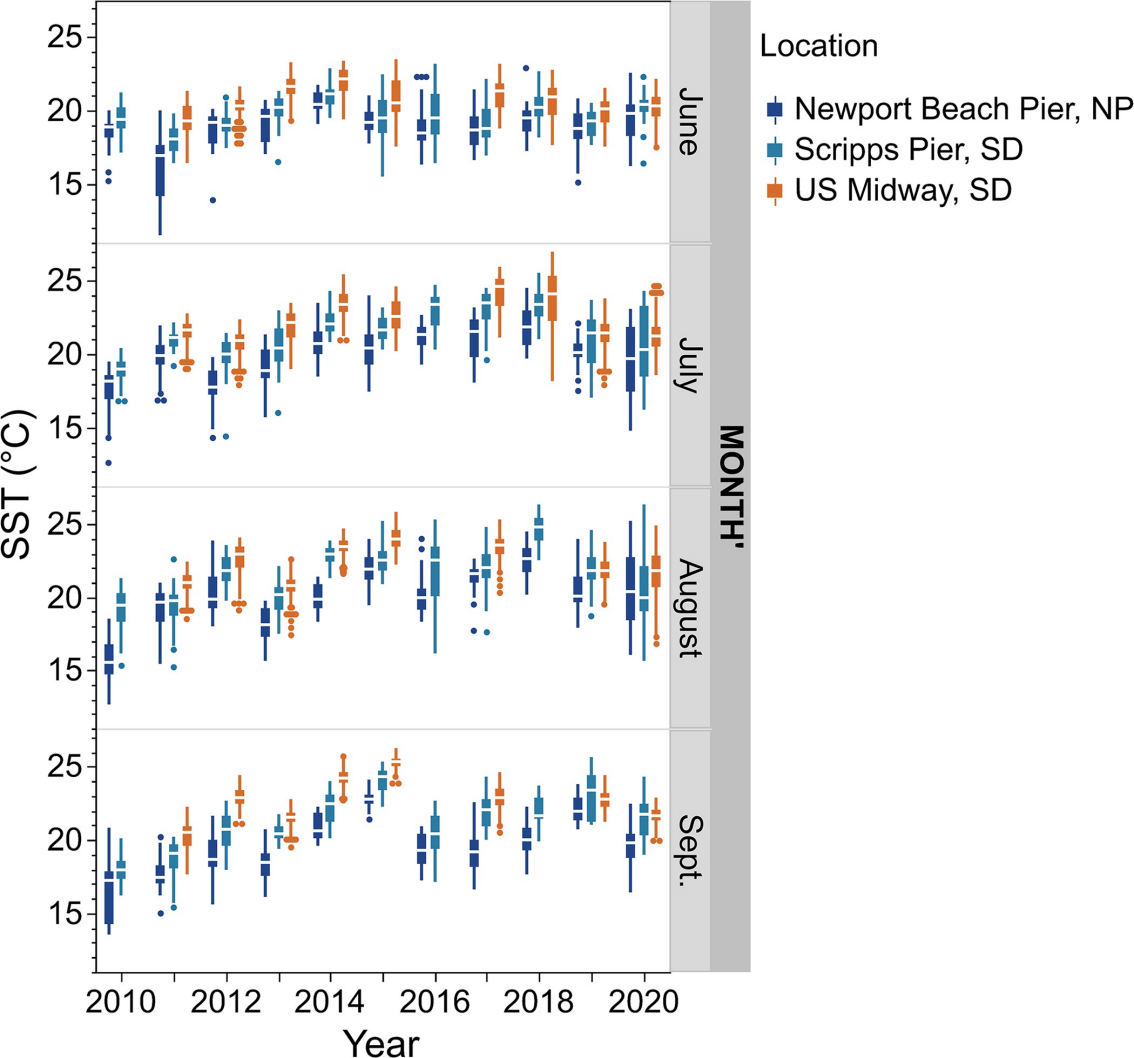
Sea Surface Temperatures for Newport (NP) and San Diego (SD) regions, CA, USA across years (2010–2020) during months June (top panel) through September (bottom panel). Dark blue = Newport Beach Pier (NP), light blue = Scripps Pier (SD), and orange = US Midway (SD). Quartiles displayed in the box plot; median is middle line; points are outliers.

Overall, median summer SST increased between 2010 and 2014 and has been consistently warmer since 2014 (Fig 4). In both Newport Bay and San Diego Bay, the coolest average monthly temperatures occurred in 2010. From 2010 to 2014 there was a clear 2–5°C shift upwards in the median monthly temperatures, most notable during the summer months of July–September (Fig 4). The greatest shifts in median water temperature occurred in August and September, with 4°C shifts in Newport Bay, and 3–5°C shifts in San Diego Bay. Between 2010–2014, Newport Bay and San Diego Bay saw an increase in the average SST (not shown) during summer months (June–Sept) of 3–4°C and 2–3°C, respectively. After 2014, median temperatures in all summer months varied among years, but were consistently warmer by ~2–3°C relative to the 2010–2014 time period.

## Discussion

The density of a globally-spread and often invasive NIS, *M*. *gigas*, increased substantially (up to 33 times) at a vast majority of sites across multiple habitat types within two major southern California estuaries during a decade where median SST increased approximately 2–4°C during summer months. The impact of this NIS on southern California estuarine communities is so far unknown, but given its potential for significant impacts elsewhere [e.g., 30], including on native oyster species [31], and its ability to substantially alter the habitat [20], these documented density increases should elevate the species’ profile among resource managers enabling decision-making about whether management actions should be implemented [49, 50].

The warmer median seawater temperatures we reported can be partly attributed to the combined effects of southward movement of the warm temperature anomaly, "The Blob", from the Gulf of Alaska [51] and an El Niño event in 2015/16. These events have been linked and implicated in an extreme warm sea surface temperature event that started in 2013 and progressed through 2016 throughout the Northeast Pacific Ocean [52]. Subsequently, Lonhart et al. [53] documented local patterns of extreme warming in 2018 in southern California locations including San Diego Bay and Tijuana River. Another persistent marine heat wave in the Northeast Pacific occurred during 2019–2020 [54]. In aggregate, these warming events contributed to nearly a decade of extreme warm seawater temperatures.

Temperature is a significant factor in marine invertebrate development [55], and as water temperatures increase, we are likely to observe local increases in the abundance of warm-water species [56]. For many sessile species that inhabit the sublittoral zone and have planktotrophic larvae, an increase in seawater temperatures may increase larvae production thereby resulting in increased local abundance and possible expansion of their distributional ranges [57]. Barry et al. [58] recorded significant shifts towards warm-water species in the invertebrate fauna of a rocky intertidal community over a 60-yr period in Monterey, CA, USA coincident with an increase in mean maximum summer temperatures by 2.2°C. Lonhart et al. [53] implicated marine heat waves with range expansions, extensions, and increases in abundances of 28 species between central California, USA and the Baja California Peninsula, MX within the same decade as our study. The non-indigenous barnacle, *Austrominius modestus*, was introduced to southern Britain but remained rare for several decades until populations grew substantially by 2007; increased temperatures during summer months may have enhanced breeding success [59]. Lastly, and most relevant to our study, Diederich et al [19] implicate warm summer waters with accelerating the invasion of *M*. *gigas* in the northern Wadden Sea.

The decadal-scale window of anomalous warming provided optimum conditions for *M*. *gigas* to spread throughout southern California. Warming temperatures positively impact *M*. *gigas* larval growth [60, 61] and settlement rates [62]. The changes in density we recorded coincident with warming waters are consistent with global increases in *M*. *gigas* recruitment and abundances that also occurred in association with warming summer water temperatures [e.g., 63]. Dutertre et al. [18] and Thomas et al. [21] noted that the expansion of *M*. *gigas* along the European Atlantic coast was attributable to warmer water temperatures during the summer months that provided optimal thermal and spawning condition, which increased the rate of successful reproduction, larval development, and recruitment. Similarly, Smaal et al. [64] attributed the establishment and expansion of *M*. *gigas* in the Oosterschelde Estuary to increased recruitment success driven by higher seawater temperatures. *Magallana gigas* abundance also increased in the Wadden Sea after a period of successful recruitment that coincided with above-average temperatures in July and August [19]. Our size frequency distribution data in aggregate suggest that southern California populations have been experiencing recruitment success throughout the decade of study but increases in the proportions of smaller size classes after 2015 at 3 of 4 sites suggest greater recruitment success in the latter part of the decade, coincident with warmer summer seawater temperatures.

With density increases come increases in the total abundance of this NIS, which can be substantial bay-wide and particularly at sites with large habitat areas. For example, CV contained the most habitat area (5,750 m^2^) of any site surveyed in this study; the 4 times mean increase in density we recorded translates to an abundance increase from 40,250 to 162,898 *M*. *gigas* individuals. Based on the error in measurement we recorded, the abundance could have ranged from a low of 22,540–57,960 in 2013 to a high of 137,828–187,967 in 2020, a 2 to 8 times change in abundance. These are likely conservative estimates of abundance change for the site because it is based upon our reported density in a constrained range of tidal elevations (necessary here to standardize among-year comparisons) where *M*. *gigas* density is substantially lower relative to upper intertidal elevations [41]. In fact, Perog et al. [65] recently provided the first bay-wide abundance estimate for *M*. *gigas* in San Diego Bay at greater than 30 million oysters, a remarkable number when considering that consistent reports of established populations of *M*. *gigas* in southern California were not recorded until 2000.

In addition to changes in thermal regime, other factors may have contributed to a lag in significant population growth from the first reported intentional plantings in the 1930’s through 2000. Exotic species are known to experience lags in population growth [66] due to a number of factors including changes in vector activity, impacts of already established invaders, biotic resistance, and genetic adaptation, among others [67]. In fact, it is possible that changes in any of a number of these other factors, including especially, changes in vector activity, may account for the increases in *M*. *gigas* density we observed over the past decade, but warmer summer seawater temperatures surely provided a favorable window of opportunity. Whatever the delay in and cause(s) for proliferation, it is clear that *M*. *gigas* is now common and abundant in southern California estuaries. The impact of this NIS is yet unclear, but Parker et al. [68] emphasize that abundance is among the most significant factors determining the impact of an invader and once a threshold is crossed, the whole structure of a community can be irreparably altered, leading to even more dramatic impacts.

It is also possible that the presence of *M*. *gigas* itself may sometimes beget more *M*. *gigas*. As *M*. *gigas* invades and establishes itself in a new habitat, its shells may alter the thermal regime [e.g., 16], hydrodynamic regime [69], and settlement induction regime [70, 71] in such a way as to create a positive feedback loop where, once this NIS becomes established, the habitat becomes more attractive for future generations of recruits. However, *M*. *gigas* density per se was not positively correlated with location-specific population growth rates. For example, densities of *M*. *gigas* at the two sites in Newport Bay with highest starting density actually increased at a lower rate (LI and AC, 2.2 and 2.7 times increases) than densities at the two sites with the lowest starting density (CI and FS, 23 and 32 times increases, Fig 2). Our study also did not record site-specific temperatures but rather used regionally-available temperature data sets (i.e., one within-bay temperature station per bay, plus a bay-adjacent open-coast temperature station) as a proxy for regional thermal conditions. An expanded focus on a suite of local conditions at each site (e.g., site-specific thermal regime, hydrodynamics, air temperatures, settlement, recruitment and mortality rates), beyond the scope of this study, could reveal the factors that controlled site-specific changes in density.

The data presented here provide a critical benchmark against which to compare future increases in abundance of *M*. *gigas*. Under future climate change, we expect increases in *M*. *gigas* abundances as their rate of proliferation increases in temperate regions [72] along with their chances of spawning and surviving [73]. Future studies should examine the relative contributions of seawater temperature, shifts in phytoplankton availability, and other factors known to affect reproductive success of this globally-spread NIS.
